# Comprehensive review of aortic aneurysms, dissections, and cardiovascular complications in connective tissue disorders

**DOI:** 10.1097/MD.0000000000036499

**Published:** 2023-12-01

**Authors:** Chukwuka Elendu, Dependable C. Amaechi, Tochi C. Elendu, Jennifer O. Ibhiedu, Augustina O. Torubiri, Osinachi K. Okoye

**Affiliations:** a University of California, Santa Cruz, USA; b Igbinedion University Okada, Nigeria; c Imo State University, Owerri, Nigeria; d Babcock University, Ogun State, Nigeria; e Niger Delta University, Bayelsa State, Nigeria; f Chukwuemeka Odumegwu Ojukwu University Teaching Hospital, Awka, Nigeria.

**Keywords:** aortic dissection, connective tissue disorders, Ehlers-Danlos syndrome, genetic mutations, Marfan syndrome, pathophysiology, prognosis

## Abstract

Connective tissue disorders, including Marfan syndrome (MS) and Ehlers-Danlos syndrome (EDS), are characterized by genetic mutations affecting connective tissue structural integrity. These disorders significantly elevate the risk of aortic dissection, a life-threatening condition. This comprehensive review delves into the intricate interplay between connective tissue disorders and aortic dissection, shedding light on the clinical features, pathophysiology, genetic underpinnings, diagnostic approaches, clinical management, associated comorbidities, and prognosis, mainly focusing on MS and EDS, while also exploring rare connective tissue disorders and forms of cutis laxa contributing to aortic pathology.

## 1. Introduction

Connective tissue disorders, encompassing diverse genetic conditions affecting connective tissues’ structural integrity, represent a complex and challenging domain in medical genetics and cardiovascular medicine.^[1]^ Within this intricate landscape, 2 conditions, Marfan syndrome (MS) and Ehlers-Danlos syndrome (EDS), have emerged as prominent protagonists. These disorders, distinguished by specific genetic mutations, usher individuals into a world where the fabric of their connective tissues undergoes profound transformation, leaving indelible imprints on various organ systems.

MS has long captivated the medical community with its distinctive skeletal features, ocular complications, and cardiovascular manifestations. The genetic underpinnings of MS, primarily rooted in mutations of the FBN1 gene, introduce a fundamental alteration in the composition of the extracellular matrix and its intricate dance with transforming growth factor-beta (TGFβ). These molecular nuances not only define the clinical features of MS but also unveil a critical vulnerability—a heightened propensity to aortic dissection.^[2]^

Ehlers-Danlos syndrome, in contrast, is a constellation of conditions, each with its unique genetic fingerprint. Vascular EDS stands out as a harbinger of aortic fragility, emphasizing the diversity within the EDS spectrum.^[1]^ Classification hinges upon specific genetic mutations affecting collagen production and tissue resilience, and vascular EDS, characterized by its association with aortic dissection, warrants particular clinical scrutiny.

This comprehensive review transcends the boundaries of these connective tissue disorders, venturing into other rare connective tissue disorders and forms of cutis laxa that conspire to precipitate aortic and arterial aneurysms and dissections.^[2]^ It is a testament to the intricate tapestry of genetic aberrations and their manifold consequences, underscoring the imperative of a nuanced understanding of these conditions.

Beyond the genetic intricacies, this review elucidates the epidemiological landscape, illuminating the increased susceptibility to aortic dissection in individuals grappling with connective tissue disorders. The pathophysiological mechanisms driving this vulnerability are meticulously dissected, emphasizing genetic mutations’ role and toll on the aortic wall resilience.^[3]^

Navigating the clinical labyrinth, this review unveils the multifaceted clinical management and treatment strategies that come into play when aortic dissection intersects with connective tissue disorders.^[4]^ From blood pressure control and heart rate management to administering antihypertensive agents and the delicate dance of surgical interventions, healthcare providers grapple with intricate decisions to safeguard patient health: genetic counseling and the contemplation of prophylactic surgery form integral components of this complex puzzle.

Comorbidities emerge as essential actors in this narrative, with associated conditions such as aortic aneurysms, aortic regurgitation, mitral valve prolapse, pulmonary complications, ocular abnormalities, and musculoskeletal manifestations sharing the stage.^[5]^ The interplay between these comorbidities and aortic dissection further complicates the clinical landscape.

## 2. Objectives

### 2.1. To explore the epidemiology

Investigate the prevalence, incidence, and demographic patterns of aortic dissection within the populations affected by connective tissue disorders, emphasizing MS and EDS.

### 2.2. To elucidate pathophysiological mechanisms

Provide an in-depth understanding of the molecular and structural alterations in connective tissues, such as those caused by FBN1 mutations in MS and collagen abnormalities in EDS, that contribute to the vulnerability of the aortic wall to dissection.

### 2.3. To analyze clinical presentations

Examine how aortic dissection manifests in individuals with connective tissue disorders, encompassing diverse clinical features and symptoms that aid in early recognition and diagnosis.

### 2.4. To detail diagnostic investigations

Present a comprehensive overview of laboratory tests, advanced imaging modalities used to diagnose aortic dissection in these high-risk populations, and the importance of genetic analysis for identifying underlying genetic mutations.

### 2.5. To discuss management strategies

Delve into the multifaceted management strategies, both medical and surgical, including blood pressure control, heart rate management, and genetic counseling, tailored to the specific needs of individuals with connective tissue disorders.

### 2.6. To address associated comorbidities

Highlight the common comorbidities, such as aortic aneurysms, aortic regurgitation, and musculoskeletal manifestations, and emphasize their management to improve overall patient outcomes and quality of life.

### 2.7. To evaluate prognostic factors

Analyze the factors influencing the prognosis of individuals with aortic dissection in connective tissue disorders, considering genetic mutations, the extent of dissection, early diagnosis, and the management of comorbidities.

### 2.8. To synthesize comprehensive insights

Bring together the diverse facets of aortic dissection in connective tissue disorders to provide a holistic understanding, facilitating improved patient care and better-informed decision-making for healthcare providers.

## 3. Review

### 3.1. Methodology

This comprehensive review on aortic dissection in the context of connective tissue disorders, mainly focusing on MS and Ehlers-Danlos syndrome (EDS), is based on a rigorous methodology that involved a systematic search of relevant literature, data extraction, and critical analysis. Below is an overview of the methodology:

#### 3.1.1. Literature Search and Inclusion Criteria.

Database selection: A systematic search was conducted across reputable medical databases, including PubMed, MEDLINE, Embase, and relevant academic journals. A primary focus was placed on English-language sources to ensure accessibility to the widest audience. English literature was the primary target due to its widespread use in scientific and medical research. However, articles of exceptional relevance in other languages were included when they provided unique insights or critical data that enriched the content.

Search strategy: A structured search strategy was developed using keywords and Medical Subject Headings (MeSH) terms. The search strategy was designed to identify studies, reviews, and clinical reports relevant to aortic dissection in the context of connective tissue disorders.

Inclusion criteria: Studies and reports that met the following criteria were considered for inclusion:

Relevance to aortic dissection in individuals with connective tissue disorders (MS, EDS, and related conditions).

Publication in peer-reviewed journals or reputable sources.

Availability of full-text articles or reports.

Studies published within the last decade (with consideration for the knowledge cutoff date of September 2021).

#### 3.1.2. Data extraction and categorization.

Study selection: The search results were screened based on titles and abstracts, followed by a full-text review of relevant articles. Articles were categorized into the following groups:

- Treatment strategies.
- Case reports.
- Observational studies.
- Epidemiological studies.
- Pathophysiological studies.

Quantitative data: The number of studies in each category was quantified to provide a comprehensive overview of the available literature. This quantification informs readers about the breadth of research available within each category.

#### 3.1.3. Demographic data and statistical methods.

The absence of substantial demographic data and statistical methods in this review is due to the nature of the paper, which is a comprehensive narrative review. Unlike systematic reviews or meta-analyses, narrative reviews primarily summarize existing knowledge and provide insights into a specific topic. They do not typically involve extensive statistical analyses or present detailed demographic data.

This review aimed to provide a broad overview of aortic dissection in connective tissue disorders, covering epidemiology, pathophysiology, clinical presentation, diagnostic investigations, management strategies, associated comorbidities, and prognosis. The emphasis was synthesizing existing information, presenting it cohesively, and comprehensively understanding the topic.

For readers seeking detailed demographic data or statistical analyses, further exploration of the primary research articles cited in this review is encouraged, as these original studies often include in-depth demographic information and statistical methods tailored to their specific research questions.

### 3.2. Marfan syndrome (MS)

#### 3.2.1. Definition and clinical features.

MS is a hereditary connective tissue disorder with diverse clinical features that transcend multiple organ systems. It is primarily characterized by:

Skeletal manifestations: Individuals with MS often exhibit remarkable height, disproportionately long limbs, joint hypermobility, and a tendency towards scoliosis or kyphosis. These skeletal abnormalities are attributed to altered fibrillin-1 (FBN1) function, a protein crucial for the structural integrity of connective tissues.^[1]^

Cardiovascular complications: The cardiovascular system bears a significant brunt in MS. The most severe and life-threatening complication is aortic dissection, where the aortic wall weakens and may tear, leading to catastrophic consequences. Common cardiac manifestations include aortic root dilation, mitral valve prolapse, and aortic regurgitation.^[2]^

Ocular involvement: Ocular features encompass myopia (nearsightedness), lens dislocation (ectopia lentis), and an increased risk of retinal detachment. These ocular manifestations are often early indicators of MS.^[3]^

#### 3.2.2. Pathophysiology.

The pathophysiology of MS centers around mutations in the FBN1 gene located on chromosome 15, which encodes for fibrillin-1. Fibrillin-1 is a critical component of microfibrils within the extracellular matrix, providing structural support to various tissues.^[6]^ In MS, mutations in FBN1 produce abnormal fibrillin-1 molecules that disrupt the integrity of microfibrils.^[4]^

One key aspect of MS pathogenesis is the interaction between mutant fibrillin-1 and transforming growth factor-beta. This cytokine regulates cellular functions, including cell proliferation and extracellular matrix synthesis.^[7]^ Abnormal fibrillin-1 molecules can sequester excessive amounts of TGFβ, leading to dysregulated TGFβ signaling and tissue weakening. This interaction contributes to developing aortic aneurysms and dissections, a hallmark feature of MS.^[5]^

### 3.3. Ehlers-Danlos syndrome (EDS)

#### 3.3.1. Definition and classification.

Ehlers-Danlos Syndrome (EDS) constitutes a heterogeneous group of hereditary connective tissue disorders characterized by a broad spectrum of clinical features.^[8]^ This diversity has led to the classification of at least 13 distinct subtypes associated with specific genetic mutations. Recognizing and distinguishing between these types is imperative for accurate diagnosis and targeted management.^[9]^

### 3.4. Clinical features

While the clinical manifestations of EDS are remarkably varied, several major types and their respective clinical features deserve particular attention:

Classical EDS (cEDS) typically presents with joint hypermobility, skin hyperelasticity, and easy bruising. Classical EDS is associated with mutations in the COL5A1 and COL5A2 genes, which encode collagen type V, a crucial component of connective tissues.^[1]^

Vascular EDS (vEDS): Vascular EDS is the most concerning type in the context of aortic and arterial aneurysms and dissections. Patients with vEDS are prone to spontaneous rupture of arteries, the gastrointestinal tract, and the uterus.^[10]^ This life-threatening subtype arises from mutations in the COL3A1 gene, responsible for collagen type III production.^[2]^

Hypermobile EDS (hEDS): Joint hypermobility, chronic joint pain, and skin hyperextensibility are prominent features of hEDS. This subtype lacks a well-defined genetic marker, making clinical assessment and symptomatology crucial for diagnosis.^[3]^

Kyphoscoliotic EDS (kEDS): Individuals with kEDS exhibit severe muscle hypotonia, joint laxity, and progressive scoliosis. It is linked to mutations in the PLOD1 and FKBP14 genes involved in collagen processing.^[4]^

Arthrochalasia EDS (aEDS): This rare subtype involves severe joint hypermobility and congenital hip dislocation. Mutations in COL1A1 and COL1A2, encoding collagen type I, underlie aEDS.^[5]^

#### 3.4.1. Genetic mutations.

EDS is fundamentally a genetic disorder; associated mutations are pivotal in connective tissue integrity.^[11]^ Mutations in various genes encoding collagen or collagen-related proteins disrupt connective tissues’ structural and functional properties, rendering them more susceptible to damage and dissection. Understanding the specific genetic mutations associated with each EDS subtype is crucial for diagnosing and researching potential targeted therapies.^[12]^

### 3.5. Other connective tissue disorders

Beyond EDS, other rare connective tissue disorders and conditions, such as cutis laxa and homocystinuria, can also predispose individuals to aortic and arterial aneurysms and dissections.^[13]^ These conditions share the common thread of connective tissue fragility, albeit through different genetic and biochemical mechanisms. Acknowledging the existence and nuances of these disorders is vital, as they broaden the spectrum of conditions that healthcare providers must consider when assessing patients at risk for aortic dissections and aneurysms.^[14]^

## 4. Aortic dissection in connective tissue disorders

### 4.1. Epidemiology

Understanding the epidemiology of aortic dissection within the context of connective tissue disorders is pivotal for recognizing the heightened risk affected individuals face.^[15]^ This section provides detailed insights into the prevalence, incidence, and demographic patterns of aortic dissection in patients with connective tissue disorders, focusing on MS and Ehlers-Danlos syndrome (EDS).

#### 4.1.1. Marfan syndrome (MS).

Prevalence: MS, an autosomal dominant connective tissue disorder, affects approximately 1 in 5000 to 10,000 individuals worldwide.^[1]^ While the prevalence is relatively low, it is essential to note that aortic dissection is a severe complication that significantly impacts patient morbidity and mortality.

Aortic dissection risk: Aortic dissection represents one of the most ominous complications of MS. In affected individuals, the lifetime risk of experiencing an aortic dissection ranges from 5% to 30%, with the highest risk occurring in the third and fourth decades of life.^[2]^

Demographic patterns: Aortic dissection in MS shows a preference for male patients and is often associated with an earlier age of onset compared to other risk factors. The propensity for aortic dissection in MS is particularly heightened in individuals who harbor specific FBN1 gene mutations.^[3]^

#### 4.1.2. Ehlers-Danlos syndrome (EDS).

Prevalence: EDS is a rare group of disorders, collectively estimated to affect approximately 1 in 5000 to 20,000 individuals, depending on the subtype.^[4]^ Vascular EDS (vEDS) is of utmost concern regarding aortic dissection among the various EDS subtypes.

Aortic dissection risk (vEDS): Vascular EDS poses the most substantial risk of aortic dissection within the EDS spectrum. Nearly 80% of individuals with vEDS experience vascular complications, including aortic dissection, by age 40.^[5]^

Demographic patterns: Aortic dissection in vEDS does not exhibit a significant gender bias and can manifest at an early age, often in the second or third decade of life. It is worth noting that vEDS tends to be more aggressive than other EDS subtypes regarding vascular complications.^[6]^

#### 4.1.3. Other connective tissue disorders.

While MS and vEDS dominate the discussions concerning aortic dissection in connective tissue disorders, it is essential to acknowledge the existence of other rare connective tissue disorders and conditions that contribute to the epidemiological landscape.^[16]^ These disorders may exhibit distinct prevalence and demographic patterns, necessitating specialized surveillance and management.

Understanding the epidemiology of aortic dissection within the context of connective tissue disorders is a fundamental step toward early recognition, proactive monitoring, and the implementation of tailored management strategies, ultimately improving patient outcomes in these high-risk populations.

### 4.2. Pathophysiology

To grasp the pathophysiology of aortic dissection within the context of connective tissue disorders, particularly MS and Ehlers-Danlos syndrome (EDS), it is essential to delve into the intricate molecular and structural mechanisms that render the aortic wall vulnerable to dissection.^[17]^

#### 4.2.1. Marfan syndrome (MS).

Altered extracellular matrix (ECM): The cornerstone of MS pathophysiology lies in mutations of the FBN1 gene, which encodes fibrillin-1, a vital component of the extracellular matrix (ECM). Fibrillin-1 forms microfibrils that scaffold and reinforce various tissues, including the aorta. Mutations in FBN1 produce abnormal fibrillin-1 molecules, disrupting the integrity of microfibrils and the ECM.^[1]^

Dysregulated TGFβ signaling: An intricate interplay emerges between mutant fibrillin-1 and transforming growth factor-beta, a cytokine involved in cell proliferation and ECM synthesis. In MS, mutant fibrillin-1 binds and sequesters excessive TGFβ, leading to dysregulated TGFβ signaling. This dysregulation affects smooth muscle cell function, impairs ECM production, and disrupts the structural integrity of the aortic wall.^[2]^

Aortic wall weakness: The combined effect of altered ECM and dysregulated TGFβ signaling weakens the aortic wall, particularly in the aortic root. This weakening predisposes the aorta to dilation and the formation of aneurysms. The compromised structural integrity, coupled with the mechanical stress exerted by hemodynamic forces, sets the stage for aortic dissection.^[3]^

#### 4.2.2. Ehlers-Danlos syndrome (EDS).

Collagen deficiencies: The pathophysiology of aortic dissection in EDS, especially vascular EDS (vEDS), is intricately linked to deficiencies in collagen type III, a major component of blood vessels and hollow organs. Mutations in the COL3A1 gene, responsible for collagen type III production, produce structurally impaired collagen.^[4]^

Weakened vascular wall: In vEDS, the defective collagen leads to a weakened vascular wall, particularly in the arteries and aorta.^[18]^ This vascular fragility increases the susceptibility to spontaneous rupture, including aortic dissection. The structural instability of collagen type III impairs the structural resilience of the aortic wall, rendering it prone to dissection even under normal hemodynamic conditions.^[5]^

Increased shear stress: The weakened vascular wall and structural abnormalities in vEDS can lead to turbulent blood flow and increased shear stress within the aorta. This heightened tension, combined with the structural vulnerability of the aorta, contributes to the development of aortic aneurysms and dissections.^[6]^

### 4.3. Clinical presentation

The clinical presentation of aortic dissection in individuals with connective tissue disorders, such as MS and Ehlers-Danlos syndrome (EDS), is characterized by a unique set of features that reflect the complex interplay between genetic predisposition and hemodynamic stressors. Understanding these presentations is crucial for timely diagnosis and intervention.^[19]^

#### 4.3.1. Marfan syndrome (MS).

Aortic root dilation: Aortic dissection often occurs in individuals with MS due to aortic root dilation. This dilation, which may be asymptomatic initially, can lead to symptoms such as chest pain, a pulsatile abdominal mass, or a sensation of fullness in the chest or upper abdomen as the aortic root expands.^[1]^

Sudden, severe chest pain: One of the hallmark features of aortic dissection is sudden, severe chest pain often described as tearing or ripping in nature. This pain can radiate to the back, neck, or abdomen and is typically unrelenting.^[2]^

Neurological symptoms: In some cases, aortic dissection in MS can lead to neurological symptoms due to the involvement of the ascending aorta and the arch vessels. This may manifest as stroke symptoms, such as weakness, slurred speech, or visual disturbances.^[3]^

Cardiovascular symptoms: Individuals with MS may experience cardiovascular symptoms, including palpitations, shortness of breath, and dizziness. These symptoms can result from aortic valve regurgitation or coronary artery involvement.^[4]^

#### 4.3.2. Ehlers-Danlos syndrome (EDS).

Sudden, excruciating pain: Aortic dissection in EDS, particularly vascular EDS (vEDS), is characterized by sudden, excruciating pain that often begins in the chest or back. This pain is typically described as severe, tearing, or ripping.^[5]^

Vascular emergencies: Individuals with vEDS are prone to vascular emergencies, and aortic dissection may present as acute limb ischemia, abdominal pain due to mesenteric artery involvement, or other ischemic complications in addition to chest pain.^[6]^

Hemorrhagic complications: Given the propensity for vascular fragility in EDS, aortic dissection can lead to life-threatening hemorrhagic complications. These may manifest as massive internal bleeding, hemothorax, or hemopericardium.^[7]^

Atypical presentations: It important to note that individuals with EDS may present with atypical symptoms due to their underlying connective tissue abnormalities. As such, a high degree of clinical suspicion is crucial for timely diagnosis and intervention.^[20]^

#### 4.3.3. Other connective tissue disorders.

In other rare connective tissue disorders, the clinical presentation of aortic dissection may vary depending on the specific condition and associated genetic mutations.^[21]^ However, some common themes include sudden, severe pain, neurological deficits if the aortic arch is involved, and vascular emergencies that can manifest in various ways.

Recognizing these clinical presentations and their unique nuances in the context of connective tissue disorders is paramount for healthcare providers to expedite diagnosis and initiate life-saving interventions promptly.

## 5. Laboratory abnormalities and investigations

Diagnosing aortic dissection in individuals with connective tissue disorders, such as MS and Ehlers-Danlos syndrome (EDS), requires a multifaceted approach that combines clinical evaluation with advanced imaging and laboratory tests. Here, we outline key laboratory abnormalities and investigations crucial for accurately diagnosing and assessing aortic dissection in these high-risk populations.^[22]^

### 5.1. Clinical assessment

Patient history: A thorough patient history should be obtained, focusing on the onset and characteristics of symptoms, risk factors, and relevant medical and family history, including any known connective tissue disorder.^[23]^

Physical examination: A meticulous physical examination is essential. Particular attention should be paid to blood pressure discrepancies between limbs, evidence of aortic regurgitation (e.g., diastolic murmur), and signs of vascular compromise, such as pulse or neurological deficits.^[24]^

### 5.2. Laboratory investigations

Complete blood count (CBC): A CBC may reveal anemia, which can occur due to blood loss from the dissection or complications such as hemopericardium or hemothorax.

Basic metabolic panel or comprehensive metabolic panel: These panels can help assess electrolyte imbalances, which may arise as complications of aortic dissection or its treatment.^[25]^

### 5.3. Imaging studies

Computed tomography angiography: Computed tomography angiography is the gold standard for diagnosing aortic dissection. It provides detailed anatomical information, including the dissection location and extent, branch vessel involvement, and complications such as aortic regurgitation.^[26]^

Magnetic resonance angiography: Magnetic resonance angiography is another valuable imaging modality, particularly in individuals who may be sensitive to radiation, such as pregnant patients. It offers high-resolution images of the aorta and surrounding structures.^[27]^

Transthoracic echocardiography: Transthoracic echocardiography can assess aortic valve function, aortic root dimensions, and the presence of pericardial effusion. While it may not visualize the dissection flap directly, it helps assess associated cardiac complications.^[27]^

### 5.4. Specialized tests for connective tissue disorders

Genetic testing: Genetic testing is essential for confirming the presence of connective tissue disorders like MS and EDS. Identifying genetic mutations can guide clinical management and screening of at-risk family members.^[28]^

Electron microscopy: In some cases, electron microscopy of skin biopsies can be performed to visualize collagen abnormalities, which may indicate EDS.^[29]^

### 5.5. Other investigations

Aortography: Although less commonly used today, aortography involves injecting contrast dye directly into the aorta to visualize the dissection. It may be considered when other imaging modalities are inconclusive.^[30]^

Lumbar puncture: In cases of suspected neurologic complications, a lumbar puncture may be performed to assess the presence of blood in the cerebrospinal fluid.^[30]^

Coagulation studies: Coagulation studies, including prothrombin time (PT) and activated partial thromboplastin time (aPTT), may be considered to assess coagulation abnormalities if bleeding or hemorrhage is a concern.^[24]^

Inflammatory markers: Elevated inflammatory markers, such as C-reactive protein and erythrocyte sedimentation rate, may be observed in some cases of aortic dissection, indicating an inflammatory response to the vascular injury.^[16]^

## 6. Clinical management and treatment strategies

Effectively managing aortic dissection in individuals with connective tissue disorders, such as MS and Ehlers-Danlos syndrome (EDS), necessitates a multifaceted approach that combines medical therapies, surgical interventions, and genetic counseling. Tailoring the management to the specific patient and the underlying connective tissue disorder is paramount for achieving favorable outcomes.

### 6.1. Medical management

Blood pressure control: One of the cornerstones of medical management is strict blood pressure control. Antihypertensive medications, such as beta-blockers (e.g., propranolol) and calcium channel blockers (e.g., verapamil), are commonly prescribed to reduce the force exerted on the weakened aortic wall, mitigating the risk of further dissection.^[11]^

Heart rate management: Controlling heart rate, often with beta-blockers, helps reduce the shear stress on the aorta by decreasing cardiac contractility and the speed of blood flow through the aortic arch.^[20]^

Pain management: Pain relief, typically achieved with analgesics or opioids, is essential for improving patient comfort during the acute phase. Non-opioid pain management strategies should be considered to minimize potential side effects and addiction risks.

### 6.2. Surgical interventions

Type A aortic dissection: Surgical intervention is often necessary in type A aortic dissection involving the ascending aorta. This typically involves aortic root replacement with a composite graft that includes a mechanical or bioprosthetic aortic valve. The Bentall procedure is a common surgical approach for these cases.^[13]^

Type B aortic dissection: In type B aortic dissection, which does not involve the ascending aorta, management is often more conservative, focusing on medical therapies to control blood pressure. However, surgical intervention may be considered in specific cases, such as those with malperfusion of branch vessels or evidence of impending rupture. Endovascular techniques, such as stent graft placement, may be used for select patients.^[14]^

Endovascular repair: In some instances, endovascular repair may be an option for selected patients with aortic dissection. This minimally invasive approach involves the placement of stent grafts to seal the entry tear, reinforcing the weakened aortic wall.

### 6.3. Genetic counseling

Risk assessment: Genetic counseling is paramount for individuals diagnosed with MS or EDS. It thoroughly assesses the patient genetic risk based on identified mutations and a detailed family history. Identifying at-risk family members and providing appropriate screening and counseling is a crucial aspect of genetic counseling.^[5]^

Family screening: Genetic counselors are pivotal in coordinating family screening efforts to identify individuals who may have inherited the genetic mutation. Early detection allows for timely intervention and risk reduction strategies.

Reproductive counseling: Patients with connective tissue disorders may seek reproductive counseling to assess the risk of passing on the genetic mutation to their offspring. Preimplantation genetic diagnosis and prenatal testing can be discussed to assist with family planning decisions.

Long-term follow-up: Genetic counseling is an ongoing process that involves long-term follow-up to monitor disease progression, adapt management strategies, and address the evolving needs of patients and their families.

### 6.4. Multidisciplinary approach

Managing aortic dissection in individuals with connective tissue disorders requires a collaborative effort involving cardiovascular surgeons, interventional radiologists, genetic counselors, and a dedicated healthcare team.^[31]^ Regular follow-up and surveillance are crucial for monitoring aortic dimensions and assessing potential complications. Individualized care plans, incorporating medical and surgical interventions, aim to optimize patient outcomes and quality of life while managing the complexities of these conditions^[23]^

## 7. Clinical manifestations and complications

In individuals with connective tissue disorders, such as MS and Ehlers-Danlos syndrome (EDS), aortic dissection is often accompanied by clinical manifestations and complications that require careful management to optimize patient outcomes. Here, we delve into these associated clinical features and complications:

### 7.1. Aortic aneurysms

Marfan syndrome (MS): Aortic aneurysms are common in MS. The structural weakness of the aortic wall, primarily due to FBN1 mutations, predisposes individuals to aortic dilation and aneurysm formation. These aneurysms can involve various aorta segments, including the ascending aorta, aortic arch, and descending aorta.^[1]^ Regular imaging surveillance is crucial to monitor aneurysm size and intervene if necessary.

Ehlers-Danlos syndrome (EDS), especially vascular EDS (vEDS): Individuals with vEDS are particularly susceptible to aortic aneurysms due to the fragility of the vascular wall. Aortic aneurysms can occur at a young age and may necessitate surgical intervention to prevent rupture.^[2]^

### 7.2. Aortic regurgitation

- Marfan syndrome (MS): Aortic regurgitation, also known as aortic insufficiency, can develop in MS due to aortic root dilation and structural abnormalities of the aortic valve. It results in the backward blood flow from the aorta into the left ventricle during diastole. This condition may require surgical correction, often with aortic root replacement.^[3]^

### 7.3. Mitral valve prolapse

- Marfan syndrome (MS): Mitral valve prolapse can occur concurrently with MS. This condition involves the displacement of the mitral valve leaflets into the left atrium during systole. Although Mitral valve prolapse may not always require intervention, it should be closely monitored for the development of mitral regurgitation.^[4]^

### 7.4. Pulmonary complications

- Marfan syndrome (MS): MS can lead to pulmonary complications such as pneumothorax (collapsed lung) and sleep apnea. Pneumothorax results from characteristic skeletal abnormalities and lung tissue fragility. Sleep apnea may be related to craniofacial features and should be addressed to improve overall quality of life.^[5]^

### 7.5. Ocular abnormalities

- Marfan syndrome (MS): Ocular manifestations, including myopia (nearsightedness), lens dislocation (ectopia lentis), and retinal detachment, are common in MS. Regular ophthalmological assessments are crucial to monitor and manage these ocular complications, which can significantly impact vision.^[6]^

### 7.6. Musculoskeletal manifestations

Marfan syndrome (MS): MS is characterized by musculoskeletal manifestations, including joint hypermobility, scoliosis, and chest wall deformities. These can lead to chronic pain and discomfort, which may require physical therapy and orthopedic management.^[7]^

Ehlers-Danlos syndrome (EDS): Musculoskeletal manifestations are a hallmark across different EDS subtypes. Joint hypermobility, joint dislocations, and chronic joint pain are common. Physical therapy and joint protection strategies are integral to managing these musculoskeletal issues and improving quality of life.^[8]^

## 8. Prognosis

The prognosis for individuals with connective tissue disorders, such as MS and Ehlers-Danlos syndrome (EDS), who experience aortic dissection, is influenced by a complex interplay of factors. Aortic dissection carries inherent risks, but timely diagnosis, genetic mutations, the extent of dissection, and the management of comorbidities all play significant roles in determining outcomes.^[32]^

### 8.1. Genetic mutations

Marfan syndrome (MS): The specific genetic mutation in MS can influence the prognosis. Individuals with specific FBN1 mutations may have a more aggressive disease course with a higher risk of aortic dissection. Genetic testing can help identify the mutation and guide risk assessment.^[1]^

Ehlers-Danlos syndrome (EDS): The prognosis of EDS is influenced by the subtype and the associated genetic mutation. For instance, vascular EDS (vEDS) carries a significantly higher risk of aortic dissection than other subtypes. Identification of the underlying genetic mutation is essential for prognosis assessment.^[2]^

The extent of dissection:

- The extent and location of the aortic dissection play a pivotal role in prognosis. Type A aortic dissections involving the ascending aorta are generally more life-threatening and often require surgical intervention. Type B dissections, which do not affect the ascending aorta, may have a relatively better prognosis but still need vigilant medical management.^[3]^

Early diagnosis:

- Timely diagnosis is paramount. Aortic dissection is a medical emergency, and swift intervention significantly improves outcomes. Delays in diagnosis and treatment can lead to complications, including organ malperfusion and rupture, which can substantially worsen the prognosis.^[4]^

Comorbidities:

- The presence and management of comorbidities, such as aortic aneurysms, aortic regurgitation, and other cardiovascular complications, substantially impact prognosis. Effectively managing these comorbidities can improve overall quality of life and extend survival.^[5]^

Medical and surgical management:

- Appropriate medical management, including blood pressure control and heart rate management, can help reduce the risk of further dissection and complications. Surgical interventions, such as aortic root replacement or endovascular repair, are often necessary and can be life-saving in many cases.^[6]^

Long-term surveillance:

- Regular follow-up and surveillance are essential for individuals who have experienced aortic dissection. This ongoing monitoring allows healthcare providers to assess aortic dimensions, evaluate the success of surgical interventions, and detect potential complications early.^[7]^

Lifestyle modifications:

- Lifestyle modifications, including avoiding strenuous activities, managing blood pressure, and addressing smoking and other risk factors, can significantly impact long-term prognosis. Lifestyle changes can help reduce the risk of further aortic events.^[8]^

## 9. Conclusion

This comprehensive review has shed light on the intricate landscape of aortic dissection in connective tissue disorders, focusing on MS and Ehlers-Danlos syndrome (EDS). The information presented underscores the importance of a holistic understanding of these conditions, encompassing epidemiology, pathophysiology, clinical presentation, diagnostic investigations, management strategies, associated comorbidities, and prognosis.

Connective tissue disorders, characterized by genetic mutations that disrupt the structural integrity of connective tissues, present a unique challenge in the healthcare landscape. Individuals affected by these disorders face a heightened risk of aortic dissection, a life-threatening condition demanding prompt diagnosis and intervention.

The epidemiological insights emphasize the prevalence and demographic patterns of aortic dissection within these high-risk populations, enabling healthcare providers to identify those at greater risk. In-depth discussions of pathophysiology elucidate the molecular and structural intricacies that render the aortic wall susceptible to dissection, facilitating the development of targeted therapies.

Clinical presentations are nuanced, and understanding how aortic dissection manifests in individuals with connective tissue disorders is instrumental for timely diagnosis and intervention. Diagnostic investigations provide a roadmap for clinicians, encompassing laboratory tests, advanced imaging, and genetic analysis to guide accurate diagnosis and tailored management.

The multifaceted management strategies, ranging from medical therapies to surgical interventions and genetic counseling, underscore the necessity of a multidisciplinary approach. Comorbidities, including aortic aneurysms, aortic regurgitation, and musculoskeletal manifestations, necessitate comprehensive care plans to optimize patient outcomes.

Lastly, the prognosis hinges on factors such as genetic mutations, the extent of dissection, early diagnosis, and effective management of associated comorbidities. Vigilant surveillance, lifestyle modifications, and ongoing medical care are pivotal in enhancing the long-term outlook for individuals living with these challenging conditions.

## Author contributions

**Conceptualization:** Chukwuka Elendu, Dependable C. Amaechi, Tochi C. Elendu, Augustina O. Torubiri.

**Data curation:** Chukwuka Elendu, Dependable C. Amaechi, Tochi C. Elendu.

**Formal analysis:** Chukwuka Elendu, Dependable C. Amaechi, Tochi C. Elendu.

**Funding acquisition:** Tochi C. Elendu.

**Investigation:** Chukwuka Elendu, Dependable C. Amaechi, Tochi C. Elendu.

**Methodology:** Chukwuka Elendu, Dependable C. Amaechi, Tochi C. Elendu.

**Project administration:** Chukwuka Elendu, Dependable C. Amaechi, Tochi C. Elendu, Augustina O. Torubiri.

**Resources:** Chukwuka Elendu, Dependable C. Amaechi, Tochi C. Elendu, Jennifer O. Ibhiedu, Osinachi K. Okoye.

**Software:** Chukwuka Elendu, Dependable C. Amaechi, Tochi C. Elendu, Augustina O. Torubiri.

**Supervision:** Chukwuka Elendu, Dependable C. Amaechi, Tochi C. Elendu, Jennifer O. Ibhiedu, Augustina O. Torubiri, Osinachi K. Okoye.

**Validation:** Chukwuka Elendu, Dependable C. Amaechi, Tochi C. Elendu, Jennifer O. Ibhiedu.

**Visualization:** Chukwuka Elendu, Dependable C. Amaechi, Tochi C. Elendu, Jennifer O. Ibhiedu, Augustina O. Torubiri.

**Writing – original draft:** Chukwuka Elendu, Dependable C. Amaechi, Tochi C. Elendu, Jennifer O. Ibhiedu.

**Writing – review & editing:** Chukwuka Elendu, Dependable C. Amaechi, Tochi C. Elendu, Jennifer O. Ibhiedu, Augustina O. Torubiri, Osinachi K. Okoye
